# Migratory routes, domesticated birds and cercarial dermatitis: the distribution of *Trichobilharzia franki* in Northern Iran

**DOI:** 10.1051/parasite/2020073

**Published:** 2021-01-12

**Authors:** Keyhan Ashrafi, Meysam Sharifdini, Abbas Darjani, Sara V. Brant

**Affiliations:** 1 Department of Medical Parasitology and Mycology, School of Medicine, Guilan University of Medical Sciences Rasht 41996-13776 Iran; 2 Skin Research Center, Department of Dermatology, Razi Hospital, Guilan University of Medical Sciences Rasht 41996-13776 Iran; 3 Museum of Southwestern Biology, Division of Parasites, Department of Biology, University of New Mexico, 1 University of New Mexico MSC03 2020 Albuquerque New Mexico 87131 USA

**Keywords:** *Trichobilharzia franki*, Iran, *Anas platyrhynchos domesticus*, Cercarial dermatitis

## Abstract

*Background*: One of the major migration routes for birds going between Europe and Asia is the Black Sea-Mediterranean route that converges on the Volga Delta, continuing into the area of the Caspian Sea. Cercarial dermatitis is a disorder in humans caused by schistosome trematodes that use aquatic birds and snails as hosts and is prevalent in areas of aquaculture in Northern Iran. Before the disorder can be addressed, it is necessary to determine the etiological agents and their host species. This study aimed to document whether domestic mallards are reservoir hosts and if so, to characterize the species of schistosomes. Previous work has shown that domestic mallards are reservoir hosts for a nasal schistosome. *Results*: In 32 of 45 domestic mallards (*Anas platyrhynchos domesticus*) (71.1%), the schistosome *Trichobilharzia franki*, previously reported only from Europe, was found in visceral veins. Morphological and molecular phylogenetic analysis confirmed the species designation. These findings extend the range of *T. franki* from Europe to Eurasia. *Conclusion*: The occurrence of cercarial dermatitis in Iran is high in areas of aquaculture. Previous studies in the area have shown that domestic mallards are reservoir hosts of *T. regenti*, a nasal schistosome and *T. franki,* as shown in this study. The genetic results support the conclusion that populations of *T. franki* from Iran are not differentiated from populations in Europe. Therefore, the schistosomes are distributed with their migratory duck hosts, maintaining the gene flow across populations with compatible snail hosts in Iran.

## Abstract (fr)

*Contexte* : L’une des principales voies de migration des oiseaux à destination et en provenance de l’Europe et de l’Asie est la route mer Noire-Méditerranée qui converge vers le delta de la Volga et se poursuit dans la région de la mer Caspienne. La dermatite cercarienne est une affection causée chez l’homme par des trématodes Schistosomatidae utilisant des oiseaux aquatiques et des mollusques comme hôtes, qui est répandue dans les zones d’aquaculture du nord de l’Iran. Avant de pouvoir lutter contre cette parasitose, il est nécessaire de connaître les agents étiologiques et leurs espèces hôtes. Ce travail vise à documenter si les canards domestiques sont des hôtes réservoirs et si oui, à caractériser les espèces de schistosomes. Des travaux antérieurs ont montré que les canards domestiques sont des hôtes réservoirs pour un schistosome nasal. *Résultats* : Chez 32 de 45 canards domestiques (*Anas platyrhynchos domesticus)* (71,1 %), le schistosome *Trichobilharzia franki*, précédemment signalé uniquement en Europe, a été trouvé dans les veines viscérales. L’identification de l’espèce a été vérifiée par une analyse morphologique et phylogénétique moléculaire. Cela étend l’aire de répartition de l’Europe à l’Eurasie. *Conclusion* : La dermatite cercarienne est répandue dans les zones d’aquaculture en Iran. Des travaux antérieurs dans la région ont montré que les canards domestiques sont des hôtes réservoirs de *T. regenti*, un schistosome nasal et de *T. franki*, comme le montre cette étude. Les résultats génétiques soutiennent que les populations de *T. franki* d’Iran ne sont pas différenciées des populations d’Europe. Par conséquent, les schistosomes se dispersent avec leur hôte canard lors de la migration, maintenant un flux génétique entre les populations avec des mollusques hôtes compatibles en Iran.

## Introduction

One of the major migration routes for birds going between Europe and Asia is the Black Sea-Mediterranean route that converges on the Volga Delta, continuing into the area of the Caspian Sea. Birds along this route migrate twice a year, nest, or stay for the winter. Therefore, the surrounding areas are visited year-round by migratory birds, particularly waterfowl. Aquaculture is a common occupation in many areas (covering five countries) around the Caspian Sea, but this type of farming is often associated with parasitic diseases [18, 47]. The water that is used for plants, ducks, fish, or crustaceans and other invertebrates is often inhabited by aquatic gastropods that can host a myriad of trematodes. Both domestic and wild mammals and birds use the water, as do humans, creating many opportunities for life cycles of several species of parasites to establish. One of these life cycles can result in a disorder called cercarial dermatitis (CD) or swimmer’s itch [41], caused by digenetic trematodes in the family Schistosomatidae. These worms have a two-host life cycle where adult worms live in a mammalian or avian host, and the intermediate host is an aquatic gastropod. The emerging free-swimming larval stages (cercariae) from the gastropod penetrate the skin of humans causing an allergic reaction that can last up to a week [43]. In an aquaculture environment, this involves the gastropods that naturally establish in water and domestic ducks, and sometimes migratory birds. Discovering the species of schistosome and their host diversity along a migratory route is a foundational step to initiating targeted control programs for CD. It is more manageable to control one duck species in the life cycle than all duck species, so more specific knowledge facilitates control or mitigation of the disease.

It is only within the last decade that there has been a concerted effort to study the epidemiology of CD in regions of the Middle East, as cases, particularly in rice fields, are gaining more attention [9, 26, 27, 30, 31, 35, 39, 51–53, 71]. Much of the work on CD in this area has been conducted in Iran, documenting the neglected status of the disease and narrowing down the critical hosts and worm species for transmission. A summary of research in Iran thus far shows that there are at least three common species of *Trichobilharzia* Skrjabin and Zakharov, 1920 that have been found in ducks and snails in Northern Iran [6, 26, 31, 51, 53, 71]. Recently, at least one species has been found in the mesenteric veins (9, 30) and a second species in the nasal tissue of its duck hosts [6, 26], particularly *Spatula clypeata* (Linnaeus, 1758) and *Anas platyrhynchos* (Linnaeus, 1758). Avian schistosomes have been recovered from the snail hosts *Radix gedrosiana* (Annandale & Prashad, 1919) and *Radix auricularia* (Linnaeus, 1758), and two species of *Trichobilharzia* from the mesenteric veins of their duck hosts [71]. The molecular identity of the avian schistosome from *R. gedrosiana* has not yet been confirmed [9, 27, 71].

Recent studies have uncovered a more detailed pattern of relationships among species of *Trichobilharzia* across a broader geographic range that encompasses the avian host migration routes [6, 25, 26, 37, 38, 61]. These patterns capture genetic diversity in the worms that reflect the long distances their bird hosts move (e.g. [25]). Additionally, finding these schistosomes in domestic or resident birds indicates that the snail host species is available, or at least a susceptible snail host (often a congener). The work herein provides more data demonstrating the significant impact of host mobility and ecology on the distribution and diversity of avian schistosomes [25]. This work aims to continue the survey and documentation of schistosomes in Iran, particularly in the rice-growing areas in the north. Additionally, the role of domestic mallards *Anas platyrhynchos domesticus* as reservoir hosts is further examined.

## Materials and methods

### Study area

This study was performed in Guilan Province of northern Iran, situated at the Western shores of the Caspian Sea (38° 28′ 58″ N, 50° 35′ 59″ E). This province consists of coastal plains, foothills and forested mountainous areas with a humid subtropical climate and the heaviest rainfall in the country. Mean rainfall in Guilan Province is about 1500 mm. The coastal plains along the Caspian Sea and near the foothills are mainly used for rice paddies, the same as that of Mazandaran Province, located to the east of Guilan Province, where cases of CD have also been reported. Guilan Province includes 2380 km^2^ of rice fields and produces about 40% of the rice products in the country. After rice harvesting from early August to late September, the rice paddies, especially those located near the farmer’s houses, become areas for livestock grazing and domestic duck breeding. The domestic mallard constitutes an important part of the diet of the indigenous population and is sold weekly at local markets. Late fall also coincides with the start of the rainy season in Guilan Province and the rice fields receive large amounts of water. These paddies then continue as suitable environments to sustain the snail intermediate hosts and maintain contact between the ducks and snails for transmission. There are also many water collections and small streams around Guilan villages, all of which have snails and sometimes ducks which widens the areas of transmission [6].

### Parasite collection

**Figure 1:**
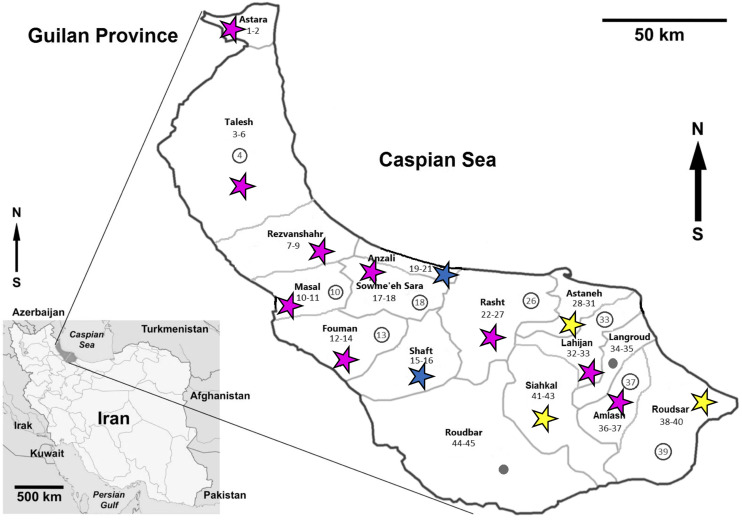
Map of Iran with Guilan province highlighted, showing collecting localities. Pink stars = provinces with positive ducks for both *T. franki* and *T. regenti*, and in some cases co-infections; Yellow stars = provinces with positive ducks for only *T. franki*; Blue stars = provinces with positive ducks for only *T. regenti*; Circles without numbers = areas with negative ducks; and Circles with numbers = localities from which worms were used for sequencing.

Domestic ducks *Anas platyrhynchos domesticus* were purchased directly from villagers’ houses surrounded by rice fields where the ducks were feeding from December 2017 through October 2018 (Fig. 1), the same ducks that were collected in [6]. Locality data were determined by GPS (Table 1). The ducks were transferred to the parasitology laboratory at the Guilan University of Medical Sciences and decapitated to examine for presence of visceral schistosomes [4]. Warm saline (40–45 °C) was injected into the liver via the hepatic portal vein as well as into different parts of liver tissue (for frozen birds tap water was used). Then, the liver was cut into small pieces in saline and transferred to a series of different mesh size laboratory sieves arranged from the largest to the smallest. The liver was then slowly crushed by hand on the upper sieve, while being washed using a trigger sprayer containing warm saline. This was done for each sieve size. The bottom sieve (106 μm) was slightly tilted and the remnants of the liver washings were collected by a plastic pipette from the lower side of the sieve, and the same process was performed for 53 μm and 25 μm sieves. For microscopic examination, a small part of the collected materials was then transferred into a glass dish with clean saline solution to dilute the material and obtain a thin layer for examination under a dissection microscope for intact adults, fragments or eggs [4]. Some of the intact worms, fragments and eggs were transferred to the microtubes containing saline for rapid morphological studies (egg and adult measurements, their micrographs), and some transferred to the microtubes containing 90% alcohol for molecular studies. All procedures performed in studies involving animals were in accordance with the ethical standards of the institution or practice at which the studies were conducted. This study was approved by the Ethics Committee of the Guilan University of Medical Sciences (IR.GUMS.REC.1398.109).

**Table 1:** Districts and coordinates of collecting localities. The duck host number matches the numbers on the collection localities in Figure 1. The results for *T. regenti* are from Ashrafi et al., 2018 since the birds examined for that study were the same as for this one.

District	Coordinates (latitude/longitude)		Duck No. *	*T. franki*	*T. regenti*	No. examined/infected for *T. franki*
Astara	38°25′11.3″ N/48°51′55.5″ E		1	P	P	2/1
	38°26′08.7″ N/48°51′46.1″ E		2	N	N	
Talesh	37°36′46.5″ N/49°03′30.1″ E		3	P	P	4/4
	37°36′46.4″ N/49°02′23.6″ E		4*	P	P	
	37°38′07.3″ N/49°02′50.7″ E		5	P	P	
	37°49′01.0″ N/48°55′10.1″ E		6	P	N	
Rezvanshahr	37°33′22.1″ N/49°09′09.8″ E		7	N	P	3/2
	37°33′24.5″ N/49°08′19.0″ E		8	P	P	
	37°32′36.7″ N/49°10′00.3″ E		9	P	N	
Masal	37°22′28.0″ N/49°09′13.5″ E		10*	P	P	2/2
	37°22′17.6″ N/49°08′31.2″ E		11	P	N	
Fouman	37°12′55.2″ N/49°18′22.8″ E		12	P	P	3/3
	37°13′01.7″ N/49°19′10.4″ E		13*	P	P	
	37°12′50.0″ N/49°18′46.1″ E		14	P	N	
Shaft	37°08′37.6″ N/49°23′27.9″ E		15	N	P	2/0
	37°08′29.9″ N/49°22′53.1″ E		16	N	N	
Sowme’eh Sara	37°17′23.4″ N/49°22′51.7″ E		17	P	N	2/2
	37°16′35.3″ N/49°22′20.0″ E		18*	P	P	
Anzali	37°25′22.8″ N/49°26′11.8″ E		19	N	P	3/0
	37°27′10.1″ N/49°35′49.9″ E		20	N	P	
	37°27′32.3″ N/49°30′38.7″ E		21	N	N	
Rasht	37°16′46.3″ N/49°45′45.4″ E		22	P	N	6/5
	37°16′16.2″ N/49°45′30.9″ E		23	P	P	
	37°16′30.2″ N/49°45′09.0″ E		24	N	N	
	37°10′50.5″ N/49°41′10.4″ E		25	P	P	
	37°11′16.7″ N/49°31′44.5″ E		26*	P	N	
	37°13′04.3″ N/49°30′34.8″ E		27	P	N	
Astaneh	37°15′46.2″ N/49°53′30.3″ E		28	P	N	4/4
	37°16′22.6″ N/49°54′30.1″ E		29	P	N	
	37°16′22.7″ N/49°54′40.3″ E		30	P	N	
	37°16′40.1″ N/49°55′38.3″ E		31	P	N	
Lahijan	37°13′06.4″ N/49°59′03.4″ E		32	P	N	2/2
	37°16′03.0″ N/50°07′17.6″ E		33*	P	P	
Langroud	37°11′11.9″ N/50°12′44.5″ E		34	N	N	2/0
	37°12′12.7″ N/50°10′30.0″ E		35	N	N	
Amlash	37°05′40.1″ N/50°11′53.9″ E		36	P	N	2/2
	37°03′37.8″ N/50°16′10.9″ E		37*	P	P	
Roudsar	37°08′34.8″ N/50°16′47.2″ E		38	P	N	3/3
	37°08′19.8″ N/50°16′37.8″ E		39*	P	N	
	37°11′29.2″ N/50°10′04.1″ E		40	P	N	
Siahkal	37°09′35.1″ N/49°52′58.3″ E		41	P	N	3/2
	37°09′35.0″ N/49°52′57.8″ E		42	P	N	
	37°09′51.6″ N/49°52′17.5″ E		43	N	N	
Roudbar	37°01′06.5″ N/49°36′46.3″ E		44	N	N	2/0
	37°00′43.2″ N/49°35′55.0″ E		45	N	N	
						**45/32**

*Numbers with an asterisk show the samples used for molecular studies; P = positive; N = negative.

### Morphological and genetic analyses

For morphological studies of adults and eggs, the intact worms, large fragments and eggs were transferred onto a glass slide, covered with a coverslip and measured (Tables 2, 3) under a microscope (Olympus BX50) equipped with a digital camera (TrueChrome Metrics, China) and Nomarski Piece (U-DICT, Olympus, Japan). The length and width of the eggs were measured, and the data analyzed in SPSS v. 22 (minimum, maximum, average, and SD). The remaining eggs, intact adults and worm fragments, if any, were transferred to microtubes containing 90% alcohol for molecular studies. Some of the collected samples (full length worms, fragments and eggs) were also kept in labeled microtubes in 90% alcohol in the Department of Parasitology and Mycology of the Guilan University of Medical Sciences as a permanent museum voucher. It is critical for the evolutionary characterization of organisms to have a permanent museum voucher [33, 59, 68].

**Table 2:** Measurements of fresh mounts of male worms from duck hosts *Anas platyrhynchos domesticus* represented as mean ± SD (min–max μm); ND = no data.

	This study		Müller & Kimming, 1994	
	*n*	Mean ± SD (min – max)	*n*	min – max (μm)
Width at esophagus (middle)	27	92.4 ± 11.9 (70 – 119)	5	120 – 130
Width at acetabulum level	7	113.4 ± 9.6 (91 – 137)	–	ND
Width after gynecophoric canal	4	73.4 ± 9.4 (63 – 83)	–	ND
Width at spatulate end	17	102.5 ± 16.3 (82 – 124)	–	ND
Oral sucker length	29	63.7 ± 5.9 (50 – 76)	5	51 – 77
Oral sucker width	29	53.1 ± 6.3 (45 – 71)	5	46 – 65
Acetabulum length	18	58 ± 9.9 (42 – 75)	5	46 – 51
Acetabulum width	18	69 ± 5.1 (58 – 77)	5	56 – 69
Acetabulum to anterior end	16	499.6 ± 46.2 (407 – 562)	5	458 – 530
Acetabulum to gut bifurcation	15	89.3 ± 14.3 (66 – 112)	5	74 – 104
Acetabulum to cecal reunion	3	512.5 ± 63.5 (385 – 547)	–	ND
Acetabulum to VSE	5	64.4 ± 14.7 (48 – 86)	–	ND
Acetabulum to gynecophoric canal	6	605.7 ± 67.3 (528 – 646)	5	495 – 550
*Vesicula seminalis externa* (VSE)	17	207.4 ± 37 (136 – 255)	–	ND
*Vesicula seminalis interna* (VSI)	12	194.6 ± 15.5 (157 – 215)	–	ND
Seminal vesicle length	4	381.3 ± 59.1 (293 – 419)	5	265 – 315
Gynecophoric canal length	30	360.5 ± 54.8 (258 – 461)	5	212 – 291
Gynecophoric canal width	40	139.5 ± 21.8 (107 – 197)	5	130 – 195
Gynecophoric canal to first testis	10	129.1 ± 16.8 (92 – 148)	–	ND
Gynecophoric canal to anterior end	14	1072.6 ± 119 (895 – 1290)	–	ND
Testis length	56	69.6 ± 10.8 (49 – 96)	5	95 – 106
Testis width	56	56.9 ± 14.8 (34 – 87)	–	ND
Number of testis	11	47.1 ± 9.9 (35 – 65)	–	ND
Ceca length	3	509.4 ± 53.7 (458 – 544)	5	680 – 705
Cecal reunion to anterior end	8	1101 ± 80 (1019 – 1179)	5	390 – 430
Cecal bifurcation to anterior end	8	451 ± 30.1 (410 – 479)	5	390 – 430
Body length	3	3915.3 ± 75 (3830 – 3971)	5	3.2 – 4.0 (mm)

**Table 3:** Measurements of fresh mounts of female worms from duck hosts *Anas platyrhynchos domesticus* represented as mean ± SD (min–max μm); ND = no data.

	*n*	This study	Müller & Kimmig, 1994	
		Mean ± SD (min – max)	min – max (μm)	
Width at esophagus (middle)	19	70.4 ± 15.9 (50 – 95)	112 – 129 μm	
Width at acetabulum level	6	88.7 ± 10.5 (68 – 98)	ND	
Width at spatulate end	8	90.6 ± 15.1 (75 – 113)	ND	
Width before spatulate end	3	56 ± 4.6 (52 – 61)	ND	
Oral sucker length	16	56.8 ± 8.1 (40 – 68)	57 – 64	
Oral sucker width	16	42.6 ± 9 (31 – 59)	46 – 55	
Acetabulum length	12	42.5 ± 7 (38 – 63)	38 – 47	
Acetabulum width	12	51.5 ± 7.1 (42 – 68)	49 – 58	
Acetabulum to anterior end	10	390.5 ± 72.8 (312 – 494)	455 – 545	
Acetabulum to gut bifurcation	5	84.4 ± 12 (75 – 98)	62 – 70	
Acetabulum to cecal reunion	2	549.5 ± 98.3 (480 – 619)	ND	
Acetabulum to ovary		ND	285 – 310	
Ceca length	3	641 ± 114.7 (537 – 764)	745 – 795	
Cecal bifurcation to anterior end	8	414.5 ± 27.6 (395 – 434)	390 – 430	
Cecal reunion to anterior end	3	1119 ± 110.5 (995 – 1207)	ND	
Egg length in uterus	2	157.5 ± 2.1 (156 – 159)	ND	
Egg width in uterus	2	41 ± 1.4 (40 – 42)	ND	
Body length	3	3559.7 ± 724.3 (3092 – 4394)	4.2 – 4.6 (mm)	
Eggs in feces	n	Length (μm)	Width (μm)	Length/width ratio
Müller & Kimming (1994)	18	205.8 ± 24.7 (155 – 250)	68 ± 8.9 (52.5 – 90)	3
Skirnisson & Kolarova (2008)	40	203 ± 27 (150–260)	69 ± 6 (57–84)	2.9
This study	99	193.9 ± 20.9 (154 – 250)	62.4 ± 9.4 (45 – 85)	3.1

For the genetic studies, genomic DNA was extracted from 90% ethanol-preserved worm fragments using a commercial kit (High Pure PCR Template Preparation Kit; Roche, Mannheim, Germany), according to the manufacturer’s recommended protocol. Primers BD1 (5′–GTCGTAACAAGGTTTCCGTA–3′) [12] and 4S (5′–TCTAGATGCGTTCGAARTGTCGATG–3′) [13] were used for amplification of a 1123 bp sequence of partial *ITS1* nuclear rDNA. Also, Cox1_SchistoF (5′–TCTTTRGATCATAAGCG–3′) and Cox1_SchistoR (5′–TAATGCATMGGAAAAAAACA–3′) primers were employed to amplify a 1250 bp sequence of the partial mitochondrial *cox1* gene [48]. PCR reaction was performed in a 30 μL reaction mixture containing 15 μL of PCR mix including 1.25 U Taq DNA polymerase, 200 μM of dNTPs and 1.5 mM MgCl2 (2 × Master Mix RED Ampliqon, Denmark), 10 pmol of each primer, and 3 μL of DNA sample. The thermal PCR profiles for the *cox1* gene included an initial denaturation step at 94 °C for 2 min followed by 35 cycles of denaturation at 94 °C for 30 s, annealing at 52 °C for 30 s and extension at 72 °C for 120 s, followed by a final extension step at 72 °C for 7 min. The PCR conditions of ITS1 gene amplification consisted of initial denaturation at 95 °C for 6 min, 30 cycles of 95 °C for 45 s, 55 °C for 60 s, and 70 °C for 1 min, followed by a final extension at 72 °C for 6 min. These PCR products were submitted to Bioneer Company (Korea) and sequenced in both directions using the same PCR primers.

### Reconstruction of evolutionary relationships

The phylogenetic relationship of the schistosomes found in this study were reconstructed using a mitochondrial gene region of partial *cytochrome oxidase* 1 *cox*1 (695 bp) and a nuclear gene region of the internal transcribed spacer regions *ITS1-5.8S-ITS2* (945 bp). Sequences were aligned by eye in Se-Al v 2.0a11 (tree.bio.ed.ac.uk). Phylogenetic analyses of the *cox*1 and ITS datasets were performed using Bayesian Inference in MrBayes [34] with default priors for the ITS genes (Nst = 6, rates = gamma, ngammacat = 4) and *cox*1 (parameters unlinked, each partition by codon had its own set of parameters; Nst = 6, rates = invgamma). The partitions by codon evolved under different rates (preset applyto = (all) ratepr = variable). Model selection was estimated using ModelTest [60]. Four chains were run simultaneously for 4 × 10^5^ generations, the first 4000 trees discarded as burn-in. The remaining trees were used to calculate a 50% majority-rule consensus tree with posterior probabilities. Outgroups used were defined by relationships from Brant and Loker [14] and Ebbs et al. [25]. The new sequences generated by this study were deposited in GenBank (accession numbers: MF945587–MF953396; MH410291–MH410297). See Table 4 for the list of specimens, references and GenBank accession numbers.

**Table 4:** Specimens used in this study.

Avian schistosome species	Snail host	Avian host	Country of origin	Identifier	GenBank *ITS*	GenBank *cox*1	Museum number*	Reference
*Trichobilharzia franki*	*Radix auricularia*		France	FORS4	HM131184	HM131197		Jouet et al. [37]
*Trichobilharzia franki*	*Radix auricularia*		France	FORS3		HM131198		Jouet et al. [37]
*Trichobilharzia franki*	*Radix auricularia*		France	STRS2	HM131176	HM131202		Jouet et al. [37]
*Trichobilharzia franki*	*Radix auricularia*		France	BERS1		HM131199		Jouet et al. [37]
*Trichobilharzia franki*	*Radix auricularia*		France	BERS2	HM131182			Jouet et al. [37]
*Trichobilharzia franki*	*Radix auricularia*		France	BERS67		HM131200		Jouet et al. [37]
*Trichobilharzia franki*	*Radix auricularia*		France	STRS4	HM131178			Jouet et al. [37]
*Trichobilharzia franki*	*Radix auricularia*		France	STRS6	HM131180			Jouet et al. [37]
*Trichobilharzia franki*	*Radix auricularia*		France	EAN77	HM131183	HM131201		Jouet et al. [37]
*Trichobilharzia franki*	*Radix auricularia*		France	RSFO1	AY795572			Ferté et al., [28]
*Trichobilharzia franki*	*Radix auricularia*		Czech Republic		AF356845			Dvorak et al. [23]
*Trichobilharzia franki*	*Radix auricularia*		Czech Republic			FJ174530		Brant and Loker [14]
*Trichobilharzia franki*	*Radix auricularia*		Great Britain	HamRa6	KJ775868		NHMUK 2014.4.25.1	Lawton et al. [45]
*Trichobilharzia franki*	*Radix auricularia*		Great Britain	HamRa7	KJ775869		NHMUK 2014.4.25.2	Lawton et al. [45]
*Trichobilharzia franki*	*Radix auricularia*		Denmark	DK1	KJ775869		ZMUC-TRE-10-12	Christiansen et al. [19]
*Trichobilharzia franki*	*Radix auricularia*		Switzerland	auri1 1100	AJ312041			Picard and Jousson [56]
*Trichobilharzia franki*	*Radix auricularia*		Switzerland	auri2 1100	AJ312042			Picard and Jousson [56]
*Trichobilharzia franki*	*Radix auricularia*		Italy		MK053632			De Liberato et al. [20]
*Trichobilharzia franki*	*Radix auricularia*		Italy		HM596077			Cipriani et al. [17]
*Trichobilharzia franki*		*Anas p. domesticus*	Iran	VT3	MF945588	MF945593	Guilan University	This study
*Trichobilharzia franki*		*Anas p. domesticus*	Iran	VT5	MF945589	MF945594	Guilan University	This study
*Trichobilharzia franki*		*Anas p. domesticus*	Iran	VT2	MF945587	MF945592	Guilan University	This study
*Trichobilharzia franki*		*Anas p. domesticus*	Iran	VT18	MF945591	MF945596	Guilan University	This study
*Trichobilharzia franki*		*Anas p. domesticus*	Iran	VT16	MF945590	MF945595	Guilan University	This study
*Trichobilharzia franki*		*Anas p. domesticus*	Iran	VR	MH410293	MH410297	Guilan University	This study
*Trichobilharzia franki*		*Anas p. domesticus*	Iran	VL	MH410292	MH410296	Guilan University	This study
*Trichobilharzia franki*		*Anas p. domesticus*	Iran	VA	MH410291	MH410295	Guilan University	This study
*Trichobilharzia* sp.	*Radix auricularia*		Czech Republic	Ra1	AY713969			Rudolfova et al. [63]
*Trichobilharzia* sp.	*Radix auricularia*		Poland	Ra2	AY713964			Rudolfova et al. [63]
*Trichobilharzia* sp.	*Radix auricularia*		Finland	F3	FJ609411			Aldhoun et al. [3]
*Trichobilharzia* sp.	*Ampullaceana balthica*		Iceland	V2	FJ469812			Aldhoun et al. [2]
*Trichobilharzia* sp.	*Ampullaceana balthica*		Iceland	FPC	FJ469820			Aldhoun et al. [2]
*Trichobilharzia* sp.	*Ampullaceana balthica*		Iceland	FPB	FJ469819			Aldhoun et al. [2]
*Trichobilharzia* sp.	*Ampullaceana balthica*		Iceland	8	FJ469816			Aldhoun et al. [2]
*Trichobilharzia* sp.	*Ampullaceana balthica*		Switzerland	ov2 1100	AJ312044			Picard and Jousson [56]
*Trichobilharzia* sp.	*Ampullaceana balthica*		Switzerland	ov1 1100	AJ312043			Picard and Jousson [56]
*Trichobilharzia* sp.	*Physa marmorata*		Brazil	HAP2013	KJ855997	KJ855996	MSB:Para:19006	Pinto et al. [57]
*Trichobilharzia* sp. Rb	*Ampullaceana balthica*		Iceland	F2IS	HM131186			Jouet et al. [37]
*Trichobilharzia* sp. Rb	*Ampullaceana balthica*		Iceland	FSIS	HM131190			Jouet et al. [37]
*Trichobilharzia* sp. Rb	*Ampullaceana balthica*		Iceland	F5ISB	HM131189			Jouet et al. [37]
*Trichobilharzia* sp. Rb	*Ampullaceana balthica*		Iceland	ICR1	HM131191			Jouet et al. [37]
*Trichobilharzia* sp. Rb	*Ampullaceana balthica*		Iceland	ls19	FJ469808			Aldhoun et al. [2]
*Trichobilharzia* sp. Rb	*Ampullaceana balthica*		Iceland	ls25	FJ469809			Aldhoun et al. [2]
*Trichobilharzia* sp. Rb	*Ampullaceana balthica*		Iceland	11	FJ469814			Aldhoun et al. [2]
*Trichobilharzia* sp. Rb	*Ampullaceana balthica*		Iceland	14	FJ469811			Aldhoun et al. [2]
*Trichobilharzia* sp. Rb	*Ampullaceana balthica*		Iceland	H	FJ469810			Aldhoun et al. [2]
*Trichobilharzia* sp. Rb	*Ampullaceana balthica*		Iceland	M2	FJ46982			Aldhoun et al. [2]
*Trichobilharzia* sp. Rb	*Ampullaceana balthica*		Iceland	ls23	FJ469805			Aldhoun et al. [2]
*Trichobilharzia* sp. Rb	*Ampullaceana balthica*		France	DOUC1		HM131205		Jouet et al. [36, 37]
*Trichobilharzia* sp. Rb	*Ampullaceana balthica*		France	EAN57	HM131194	HM131204		Jouet et al. [37]
*Trichobilharzia* sp. Rb	*Ampullaceana balthica*		France	EAN79	HM131196			Jouet et al. [37]
*Trichobilharzia* sp. Rb	*Ampullaceana balthica*		France	EAN30	HM131192			Jouet et al. [36, 37]
*Trichobilharzia* sp. Rb	*Ampullaceana balthica*		France	EAN31		HM131203		Jouet et al. [36, 37]
*Trichobilharzia* sp. Rb	*Ampullaceana balthica*		Norway	TFPTAK4	KY513273		HCIP D-735–D-750	Soldanova et al. [67]
*Trichobilharzia* sp. Rb	*Ampullaceana balthica*		Norway	TFPTAK1	KY513270		HCIP D-735–D-750	Soldanova et al. [67]
*Trichobilharzia* sp. Rb	*Ampullaceana balthica*		Norway	TFPTAK3	KY513272		HCIP D-735–D-750	Soldanova et al. [67]
*Trichobilharzia* sp. Rb	*Ampullaceana balthica*		Norway	TFPTAK2	KY513271		HCIP D-735–D-750	Soldanova et al. [67]
*Trichobilharzia* sp. Rb	*Ampullaceana balthica*		Switzerland	ov4 1100	AJ312046			Picard and Jousson [56]
*Trichobilharzia* sp. Rb	*Ampullaceana balthica*		Switzerland	ov3 1100	AJ312045			Picard and Jousson [56]
*Trichobilharzia* sp. Rb	*Lymnaea stagnalis*		Czech Republic	LS1	AY713973			Rudolfova et al. [63]
*Trichobilharzia* sp. Rb	*Radix auricularia*		Poland	Ra3	AY713966			Rudolfova et al. [63]
								
*Trichobilharzia* sp. A		*Mareca americana*	USA	W213	FJ174570	FJ174526	MSB:Para:18646	Brant and Loker [14]
*Trichobilharzia* sp. A		*Mareca americana*	USA	W192	FJ174572	FJ174471	MSB:Para:18609	Brant and Loker [14]
*Trichobilharzia* sp. A		*Mareca americana*	USA	W182	FJ174573	FJ174525	MSB:Para:18574	Brant and Loker [14]
*Trichobilharzia* sp. A		*Mareca americana*	USA	W149		FJ174524	MSB:Para:18585	Brant and Loker [14]
								
*Trichobilharzia* sp. B		*Mareca americana*	USA	W210	KP788772		MSB:Para:18643	Ebbs et al. [25]
*Trichobilharzia* sp. B		*Mareca americana*	USA	W205	KP788770	FJ174528	MSB:Para:18638	Ebbs et al. [25]
								
*Trichobilharzia* sp. C		*Lophodytes cucullatus*	USA	W173		FJ174529	MSB:Para:18562	Brant and Loker [14]
*Trichobilharzia* sp. C		*Aix sponsa*	USA	W174		KJ855996	MSB:Para:18563	Ebbs et al. [25]
								
*Trichobilharzia physellae*	*Physa parkeri*		USA	W234		FJ174520	MSB:Para:18656	Brant and Loker [14]
*Trichobilharzia physellae*		*Aythya affinis*	USA	W171	FJ174564		MSB:Para:18565	Brant and Loker [14]
*Trichobilharzia physellae*		*Aythya affinis*	USA	W212	FJ174563		MSB:Para:18645	Brant and Loker [14]
*Trichobilharzia physellae*		*Aythya affinis*	USA	W193		FJ174518	MSB:Para:18610	Brant and Loker [14]
*Trichobilharzia physellae*		*Aythya collaris*	USA	W194	FJ174566	FJ174517	MSB:Para:18611	Brant and Loker [14]
*Trichobilharzia physellae*		*Bucephala albeola*	USA	W255	FJ174561	FJ174514	MSB:Para:19159	Brant and Loker [14]
*Trichobilharzia physellae*		*Clangula hyemalis*	USA	W211		FJ174516	MSB:Para:18644	Brant and Loker [14]
*Trichobilharzia querquedulae*	*Physa gyrina*		USA	W413	HM125959		MSB:Para:186	Brant et al. [15]
*Trichobilharzia querquedulae*		*Spatula discors*	USA	W156	FJ174554	FJ174502	MSB:Para:18590	Brant and Loker [14]
*Trichobilharzia querquedulae*		*Spatula discors*	USA	E45	FJ174555	FJ174510	MSB:Para:24778	Brant and Loker [14]
*Trichobilharzia querquedulae*		*Spatula cyanoptera*	USA	W180		FJ174505	MSB:Para:18573	Brant and Loker [14]
*Trichobilharzia querquedulae*		*Spatula clypeata*	USA	W135	FJ174557	FJ174497	MSB:Para:183	Brant and Loker [14]
*Trichobilharzia querquedulae*		*Spatula clypeata*	USA	W203	FJ174552	FJ174508	MSB:Para:18636	Brant and Loker [14]
*Trichobilharzia querquedulae*		*Spatula clypeata*	Canada	W345		FJ174509	MSB:Para:18626	Brant and Loker [14]
*Trichobilharzia querquedulae*		*Spatula rhynchotis*	New Zealand	TshovNZ	KP788760	KU057183	MSB:Para:20794	Ebbs et al. [25]
*Trichobilharzia querquedulae*		*Spatula rhynchotis*	New Zealand	W703		KU057181	MSB:Para:20792	Ebbs et al. [25]
*Trichobilharzia querquedulae*		*Spatula smithii*	South Africa	W650	KP788765		MSB:Para:18990	Ebbs et al. [25]
*Trichobilharzia querquedulae*		*Spatula smithii*	South Africa	W664		KU057180	MSB:Para:19000	Ebbs et al. [25]
*Trichobilharzia querquedulae*		*Spatula platalea*	Argentina	W833		KU057184	MSB:Para:23180	Ebbs et al. [25]
								
*Trichobilharzia regenti*	*Ampullaceana balthica*		France	EAN9		HM439499		Jouet et al. [36, 38]
*Trichobilharzia regenti*		*Mergus merganser*	France	HAR1		HM439501		Jouet et al. [38]
*Trichobilharzia regenti*		*Cygnus olor*	France	CYA18		HM439500		Jouet et al. [38]
*Trichobilharzia regenti*		*Anas platyrhynchos*	France	BERS58		HM439502		Jouet et al. [38]
*Trichobilharzia regenti*		*Anas platyrhynchos*	Iceland	AC122		HM439503		Jouet et al. [38]
*Trichobilharzia regenti*		*Anas platyrhynchos*	Iceland	AC125		HM439504		Jouet et al. [38]
*Trichobilharzia* cf. *regenti*		*Spatula clypeata*	Iran	T39		KR108325		Fakhar et al. [26]
*Trichobilharzia* cf. *regenti*		*Spatula clypeata*	France	JIT11		HM439505		Jouet et al. [38]

## Results

### Morphological identification and molecular characterization

**Figure 2:**
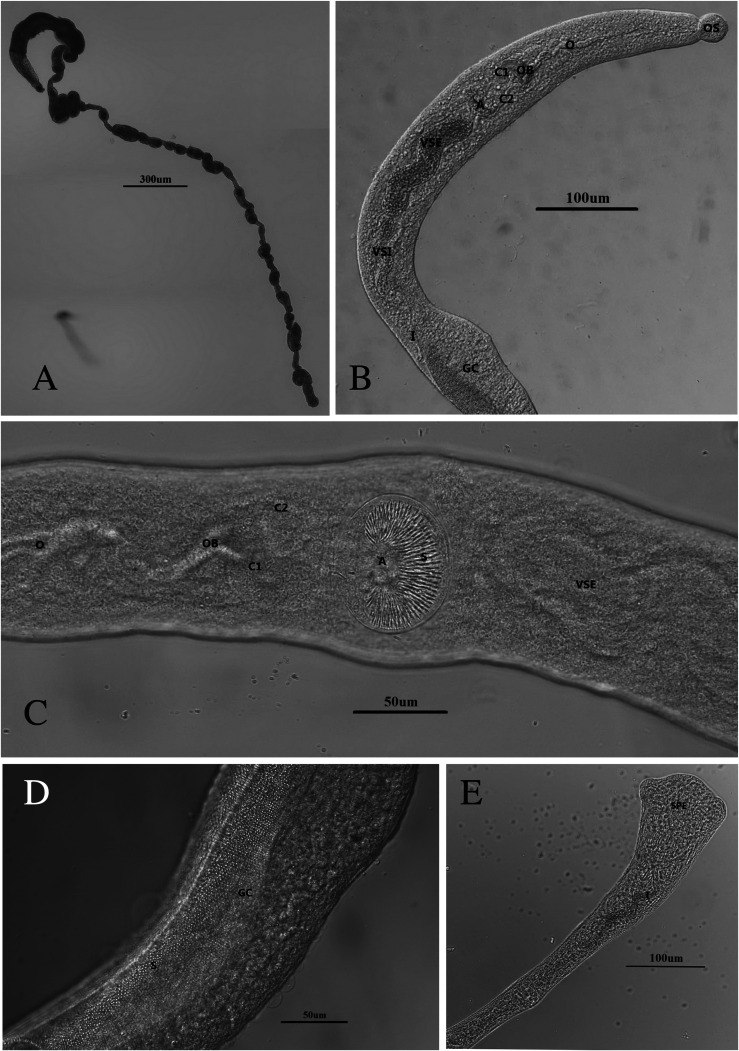
Images of an adult male worm in saline. a) full length male; b) anterior end OS = oral sucker, O = esophagus, OB = esophagus bifurcation, C1/C2 = cecum1 and cecum 2, A = acetabulum, VSE = external seminal vesicle, VSI = internal seminal vesicle, I = intestine, and GC = gynecophoric canal; c) spines on the acetabulum, codes the same as defined in (b); d) fine spines in the gynecophoric canal; e) posterior end of worm showing spatulate tail.

**Figure 3:**
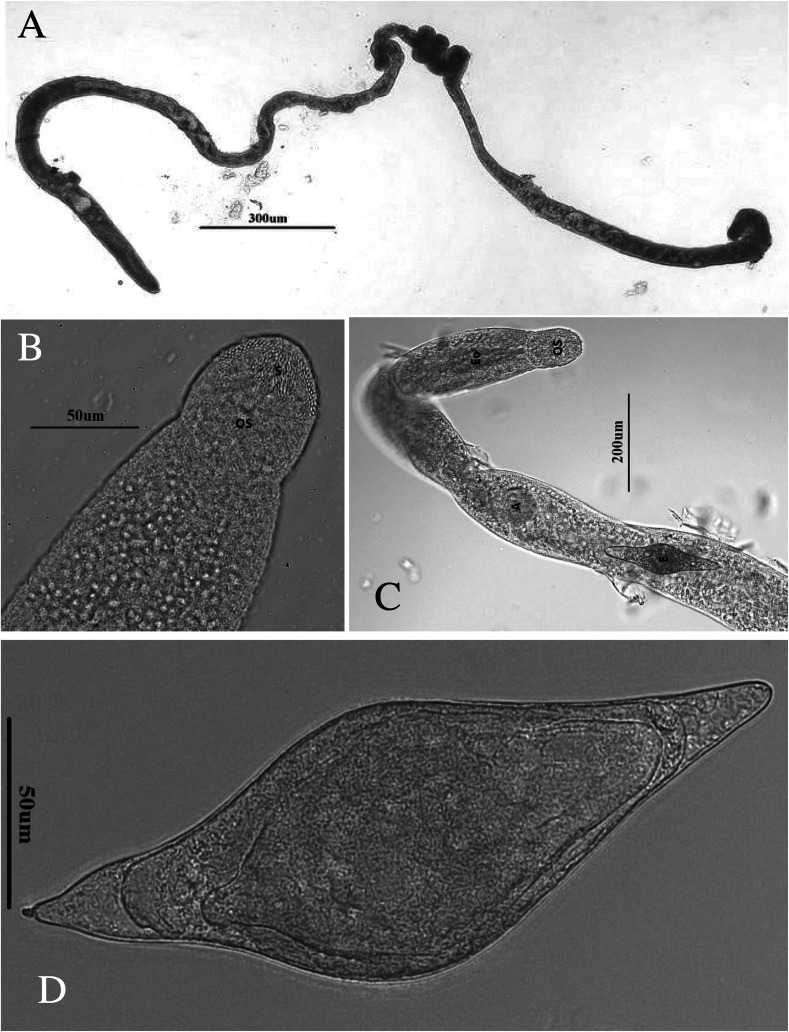
Images of an adult female worm in saline. a) full length female; b) oral sucker showing fine spines; c) anterior end showing single egg in the uterus, OS = oral sucker, OE = esophagus, A = acetabulum, E = egg; d) egg from liver washings.

From our collection of 45 ducks from 45 localities in 16 districts, *Trichobilharzia franki* Müller and Kimmig, 1994 was found at 32 sites in 12 districts; worms were found in the liver of 32/45 ducks, with 71.1% prevalence (Table 1). Because this study continues the efforts of Ashrafi et al. [6], some of these ducks were infected with both *T. franki* and the neuropathic nasal species, *T. regenti.* There were 32/45 infected with *T. franki,* 17/45 infected with *T. regenti*, and 13/45 co-infected with both species (Table 1; [6]). These worms aligned morphologically with those of the original description by Müller and Kimmig (1994) of *T. franki* derived by infections of domestic dwarf mallards with cercariae from wild collected *Radix auricularia* [54]. However, some of the measurements herein were smaller. One explanation might be because Müller and Kimmig [54] put the host tissue in a trichinelloscope, which flattens the tissue to expose the live worms; their measurements might be larger with this type of preparation method. The authors even state that the measurements should not be regarded as absolute values [54]. In addition to the measurements (Tables 2 and 3) there were other features in common with the original description. Müller and Kimmig (1994) [54] found worms mostly in the veins of the liver, but in some cases, they found worms in the gut mucosa. If worms were found in the mesenteric blood vessels, they were localized near the outer wall of the intestine and were irregular in density from the duodenum to the cloaca. In the female and male worms, the oral sucker and acetabulum are spined (Figs. 2B, 2C and 3B) and in the males the gynecophoric canal is spined (Fig. 2D), but no body spines were observed. Other similarities: cecal reunion was observed between the posterior seminal vesicle and anterior gynecophoric canal and the tail is wide, spatulate and tri-lobed (Fig. 2E). Eggs in both studies are spindle-shaped with a straight longitudinal axis with one end rounded and the other end slightly less rounded, but ending in a small spine (Fig. 3D). The uterus contained only one egg at a time (Fig. 3C), and the rounded end was pointed consistently anteriad. Males and females were similarly sized in length, as was also found in Müller and Kimmig [54]. We have morphological adult comparisons only for the original description. Any specimens included in the gene trees were from fragments of adults, eggs, or cercariae, and thus no morphological descriptions are available. Jouet et al. [37] included a description of the *T. franki* cercariae from *R. auricularia* and those from *Ampullaceana balthica* (Linnaeus, 1758); the latter is larger than *T. franki*.

**Figure 4:**
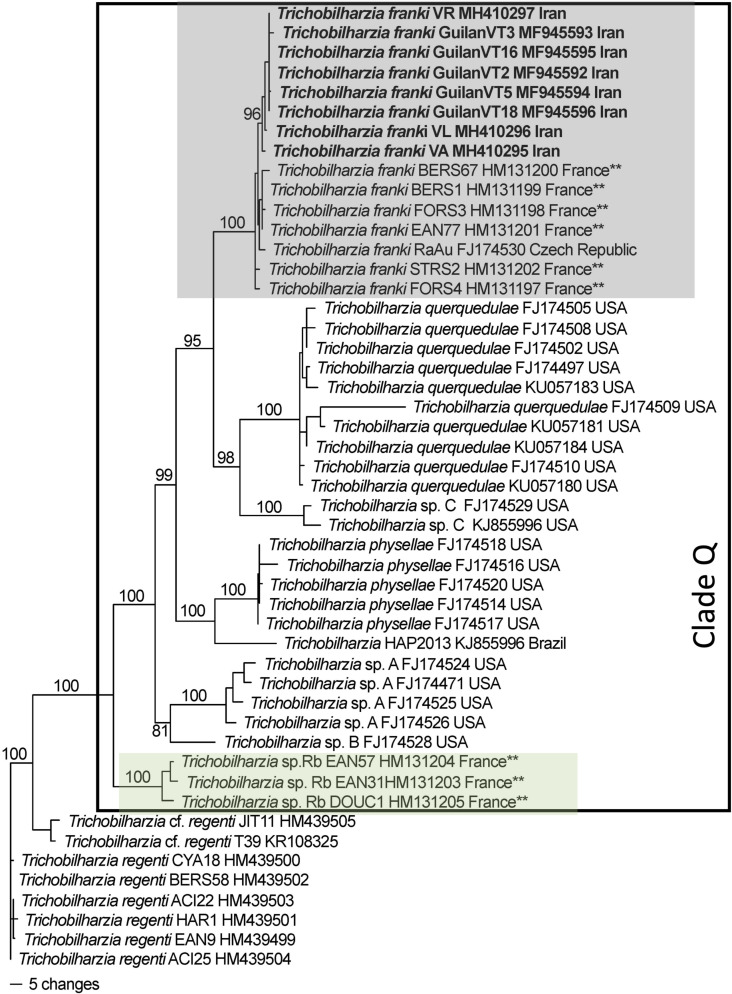
Phylogenetic tree based on Bayesian analysis of *cox1* with nodal support indicated on the branches by posterior probabilities. The outer box defines taxa in Clade Q *sensu* Brant and Loker [14]. The gray boxed clade includes individuals of *Trichobilharzia franki* with the samples from this study in bold, all other samples are from *R. auricularia* snails. The green box highlights the “*peregra*” group (*sensu* [37]); these species are often confused for *T. franki* but mostly come from *A. balthica*. The double asterisk indicates that the snail host in the study was also characterized genetically. Blue arrows indicate schistosomes from *R. auricularia* but did not group within the clade for *T. franki*. Taxa are listed with their corresponding GenBank accession number, followed by the country of collections (see Table 4).

**Figure 5:**
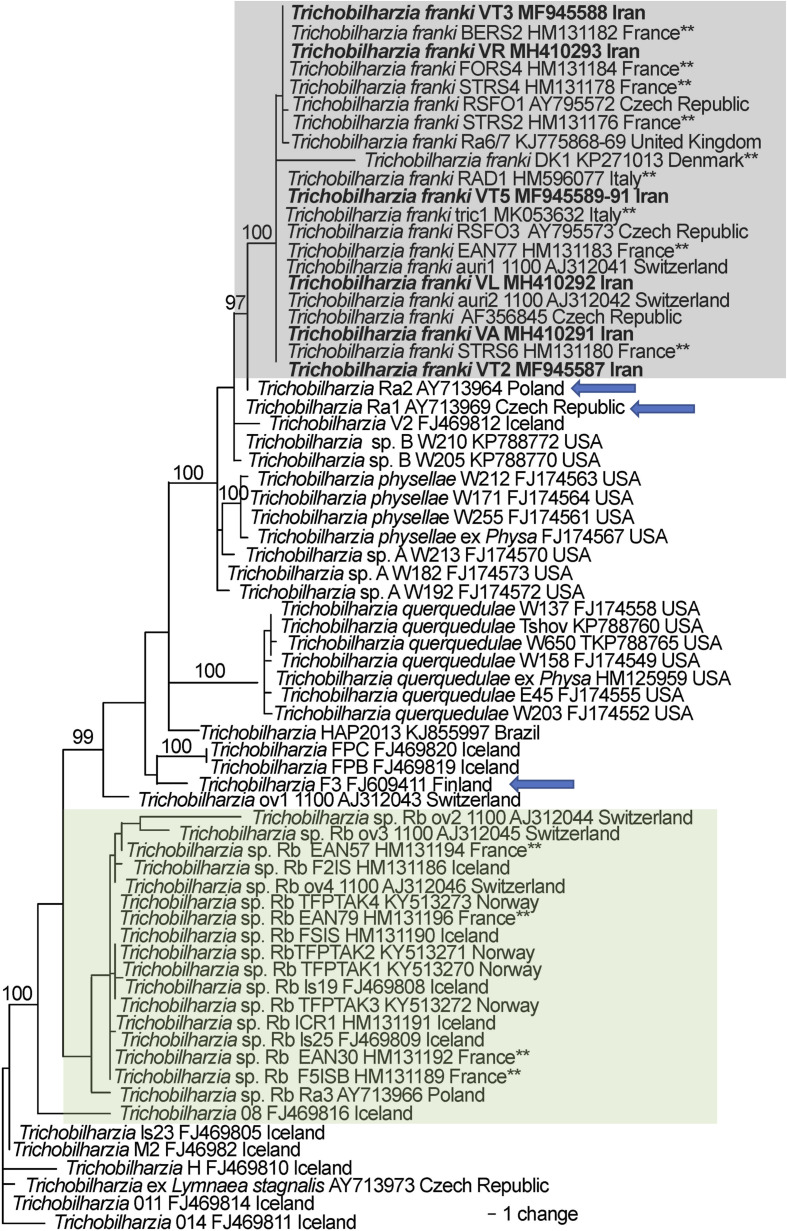
Phylogenetic tree based on Bayesian analysis of *ITS1-5.8S-ITS2* with nodal support indicated on the branches by posterior probabilities. The gray boxed clade includes individuals of *Trichobilharzia franki*, all samples from snails are from *Radix auricularia* and the samples from this study are in bold. Blue arrows point to specimens that were reported from putative *R. auricularia* outside of the *T. franki* clade. The green box highlights the “*peregra*” group (*sensu* [37]); these species are often confused for *T. franki* but mostly come from *A. balthica*. The double asterisk indicates that the snail host in the study was also characterized genetically. Taxa are listed with their corresponding GenBank accession number followed by the country of collections (see Table 4).

The phylogenetic analysis of both the *cox*1 (Fig. 4) and *ITS1-5.8S-ITS2* (Fig. 5) datasets placed the samples from this study within specimens described as *T. franki* from *Radix auricularia* snail intermediate hosts. Some of the previous studies that submitted sequences to GenBank labeled as *T. franki* were not monophyletic and most of those sequences belong to an undescribed species *Trichobilharzia franki* haplotype “*peregra*” (*sensu* [37] and were referred to as *Trichobilharzia* sp. Rb from *A. balthica* snail intermediate hosts. Furthermore, there were many haplotypes labeled *T. franki* that did not group with any previously defined clade. The clades of *T. franki* and *Trichobilharzia* sp. Rb were also found by Soldanova et al. [67]. Using uncorrected *p*-distances as a measure of genetic diversity and as a proxy for species differentiation, *T. franki* specimens from Iran were not very divergent from the available specimens of *T. franki* from GenBank. The average within species diversity was 0.1% for *ITS* and 0.7% for *cox*1, which is consistent with other species of *Trichobilharzia* (Table 5).

**Table 5:** Average pairwise uncorrected *p-*distances among taxa in the phylogenetic trees.

	ITS1 (%)	*cox*1 (%)
Intraspecific variation		
*Trichobilharzia franki*	0.1	0.7
*Trichobilharzia* sp. Rb	0.3	0.6
*Trichobilharzia querquedulae*	0.2	0.3% – 1.6% 2.4%
*Trichobilharzia physellae*	0.4	0.7
Interspecific variation		
*T. franki* – *Trichobilharzia* sp. Rb	2.7	11.5
*T. querquedulae* – *T. franki*	2.6	9.2
*T. querquedulae* – *T. physellae*	1.9	10.2
*T. physellae* – *T. franki*	1.2	10.6
*T. physellae* – *Trichobilharzia* sp. Rb	2.5	11.5
*T. querquedulae* – *Trichobilharzia* sp. Rb	3.7	11.1

## Discussion

Our results show that *T. franki*, a species of avian schistosome that occurs in the visceral veins of its anatid hosts, can be found in domestic ducks in Iran. Duck breeding is a routine activity in almost all rural areas and towns in the flatlands and foothills of Guilan Province, as well as other provinces such as Mazandaran [6, 9, 26]. In these areas, *Radix* spp. and *Physa* sp. snail are also found since they also do very well in these modified habitats. It can be assumed that with 71% prevalence in ducks found in this study, *T. franki* is a common species maintained over time and space by the ubiquity of the intermediate host in the same modified aquaculture habitats as well as the wide distribution and use of domestic mallards, an ideal reservoir host. In addition, the ducks examined here were the same ducks examined in Ashrafi et al. [6] for the neuropathogenic species *T. regenti.* An interesting question to consider is whether the diversity of *Trichobilharzia* in domestic mallards over time would reflect the diversity of schistosome species found in migratory birds that also cycle through the commonly available snail intermediate hosts? Or given the prevalence of *T. franki* and *T. regenti,* are they well established enough in a domestic life cycle that finding the rarer species from migratory birds would be difficult, or competition in either or the snail or duck hosts? Mallards are listed to host at least 10 named species (7 *Trichobilharzia* spp.) of schistosomes. Some adult schistosome species are only known from experimental infections of domestic mallards using cercariae from captured wild snails, such as *T. franki*. Distinguishing between wild mallards and resident mallards is not often defined in most papers. It is known that mallards can host schistosomes, and certainly they have made excellent experimental hosts, but it is difficult to ascertain the distribution and diversity of schistosomes in wild mallards, or if the diversity reflects what can be found in the co-occurring anseriforms.

Until now, *T. franki* had been confirmed genetically only in Europe to include mostly northern countries (France, Great Britain, Denmark, Switzerland, Czech Republic, Austria, western Russia) [3, 16, 17, 19, 20, 23, 28, 32, 36–38, 42, 45, 61, 62] and now the geographic range is extended to include Europe and Western Asia (Iran). This is the first study to find intact adults of *T. franki* and characterize them since the original description. Previous reports were based mostly on cercariae, and some on adult fragments or eggs. *Trichobilharzia franki* was first described from southwestern Germany by Müller and Kimmig (1994) from wild *Radix auricularia* snail hosts, then cycled experimentally through domestic mallards to obtain the adult worms. Since 1994, there have been few confirmed reports of adults other than small fragments or eggs, and very little effort has been made to sequence more than the nuclear *ITS*, particularly *ITS2* (which is not a useful marker for congeners) and little effort to voucher the specimens or snail hosts. There are very few mitochondrial sequences available for *T. franki*, most of which have come from cercariae in France (one from the Czech Republic and Austria), making it difficult to characterize genetic diversity across time, space and hosts, with few exceptions [37, 61]. This study has expanded geographic sampling and suggests that *T. franki* populations are not isolated, at least spanning from France to Iran. The results herein also suggest that if *Radix auricularia* (or closely related permissive species of *Radix*) and ducks use the same water body, then it is likely to find *T. franki* along the migratory route of these birds. Some of these ducks will migrate to northern Africa and if there is *R. auricularia* or perhaps another permissive snail, then likely this schistosome can extend all along the migration route. Migration was also suggested as the cause of haplotype sharing in *T. franki* from the UK and Austria [45, 61].

The question remains, how do you designate a species as *T. franki* in the absence of adult worm morphology and genetic identification of the snail host? Certainly, by genetic comparisons, other named species can mostly be eliminated as the gene trees show them grouping to the exclusion of others with strong support. However, as of this writing, GenBank does have sequences vouchered as *T. franki*, but they are the ITS2 region which is not informative for species discrimination from Vietnam (MT892757, MT895500, MT919390–MT919394), and Russia and Belarus [49, 62]. For the above sequences, the authors stated that the cercariae were recovered from putative *R. auricularia* but there is no genetic confirmation of this identification or even a morphological justification. The tree presented in Figure 3 of reference [62] highlights their samples with other putative samples of *T. franki.* However, there is no monophyletic clade of *T. franki* and their clade includes other species of Trichobilharzia making it impossible to assign a species based only on their gene tree. Unfortunately, the tree presented in Lopatkin et al. [49] did not include samples from GenBank for comparison and even though they collected cox1 data, it was not vouchered in any publicly available sequence repository. At least for the snails from Russia and Belarus, likely the snail identifications are correct since it is expected that the snail can be found in these geographic areas. The status of the GenBank record from Vietnam is unclear and the sequences currently are not featured in a publication

The natural definitive hosts of *T. franki* are not well known. There are a few short 28S sequences (552 bp) available in GenBank from duck hosts (FJ793813–FJ793818, FJ793820–FJ793822), and of these, the ones that form a clade with *T. franki*, are from *Anas platyrhynchos* and one *Cygnus olor* (Gmelin, 1789). The other duck hosts reported are *Anas crecca* (Linnaeus, 1758), whose schistosomes did not group with any clade, and *Aythya ferina* (Linnaeus, 1758), whose schistosomes grouped with the *Trichobilharzia franki “peregra”* group (*sensu* [37] and see [66]; *Trichobilharzia* sp. Rb herein), a clade that is not closely related to *T. franki* (Figs. 4 and 5, Table 5). However, eggs were not found in *Ay. fuligula* or *A. crecca* hosts; therefore it difficult to know whether worms would produce offspring or if these are dead-end hosts [37]. The occurrence of *T. franki* is likely facilitated by the widespread intermediate snail host, species of *Radix* (likely *R. auricularia*) plus the long distance migration of the anatid hosts. While the snail host in Iran is not yet known, different species of Lymnaeidae, *Galba shiraziensis* (Küster, 1862 [44]), *Stagnicola palustris*, *Radix auricularia* and *Radix* sp. have been reported in Guilan Province and are potential hosts [5, 7] for *T. franki*. There is some evidence that *R. auricularia* (= *L. gedrosiana*) is an intermediate host of *Trichobilharzia* spp. in Iran [9, 30, 31] but more studies are needed to confirm this hypothesis.

The systematics and taxonomy of the identity and distribution of *R. auricularia* are not well understood. Recent studies using more variable gene regions (*cox1*) have shown that this species may include a complex of clades [46, 70]; however, no samples from the Middle East region were included. Furthermore, it has been suggested that *R. auricularia* might be an invasive snail and/or more widespread than previously thought, but this proposition has not yet been tested [8, 40, 46]. It appears that *R. auricularia* likely plays a major role in transmission of avian schistosomes in the country, but the species has not yet been verified and unidentified species of *Radix* have yet to be characterized. Furthermore, Aksenova et al. [1] suggest that *Radix euphratica* may be more widespread in the area and Vinarski et al. [69] suggest *R. gedrosiana* should be synonymized under *R. euphratica*. The first sequences to be described as *T. franki* are from *R. auricularia* from Switzerland [56], but there is no mention in the paper about what they based their species identification on, other than that the original description from *R. auricularia* hosts [6]. This assumption of host specificity might be reasonable given that the molecular results have shown over time that cercariae from *R. auricularia* most often group with haplotypes named *T. franki* (see some clades from [3, 63] and Figs. 4 and 5 herein), but *T. franki* had not been sampled widely with genetic confirmation of their snail hosts. At least in the *ITS* tree, not all samples from *R. auricularia* form a clade (see blue arrows in Fig. 5) and many of them from *Ampullaceana balthica* (= *Radix peregra, = R. ovata*) form a clade [1, 37]). Brant and Loker [14] suggested that *T. franki* might be found in North America, but the results for both *cox*1 (Fig. 3; FJ174528) and *ITS* (Fig. 4; KP788772, KP788770) show a position outside *T. franki*. The *ITS* tree shows a grouping with a haplotype from *R. auricularia* from Poland (Fig. 5; Table 4; AY713969).

Much of the genetic diversity of *Trichobilharzia* lineages in Clade Q (*sensu* [14]; Fig. 4) that includes *T. franki* from GenBank sequences is represented by *ITS* sequences, and many of these samples do not form clades (Fig. 5). This might suggest that more diversity is yet to be discovered. Processes that may contribute to our understanding of diversity in *Trichobilharzia* that emerges from sequencing surveys include the following: (A) Incomplete lineage sorting – the *ITS* tree is based only on a nuclear region, which may have a slower mutation rate relative to a faster evolving gene, thus ancestral polymorphisms are retained, or the ancestral population was large and thus takes more time. Also, it could be that speciation within at least Clade Q has been relatively recent and nuclear copies do not match mitochondrial gene trees or species trees. However, in general, most sequences fall into taxa that group according to species (or lineages if only cercariae) and there is little or no evidence of widespread mito-nuclear discordance. (B) Hybridization – within avian schistosomes, hybridization has not been studied. However, mito-nuclear discordance is not reliable evidence of hybridization (see [58]) but a more variable gene certainly helps in diversity characterizations and species delineations. It is impossible to obtain mitochondrial or genomic data to explore hybridization or any other question with the individual worms available currently in GenBank, because there are no museum vouchers for re-evaluation. The few specimens that were vouchered are not available for destructive sampling and thus it is strongly recommended that vials of adult and cercariae (and hosts, particularly gastropods) are deposited so that we have a record of the past and material available for new investigations. (C) Host-induced variation – though poorly understood, host-induced variation can contribute to morphological variability, but it is not known how much it would affect genetic diversity in schistosomes, and most studies have used morphology, not genetics in this context, with adult worms (see [10, 65]). (D) Ecological speciation is plausible given that the offspring of the worms in the migratory hosts are being distributed along the route, at each locality the miracidia might be exposed to snails that are normally compatible but might also be exposed to novel putatively susceptible snails. For parasites, this is akin to host switching events. For a high-quality review see [64]. (E) Poor host taxonomy. One of the consequences of genetic characterizations is that it has provided a yardstick to define in more detail lineage diversity, which may not be reflected in morphological diversity. Invertebrates in particular have fallen in this category as they can often have little variation to compare and some of the observed variation is subject to change based on a myriad of abiotic and biotic influences (such as parasitism, temperature, water chemistry, etc.) rather than phylogeny. Gastropods in particular have been shown to be more species-rich than previously considered based on morphology [1, 29]. If every genetic report of *Trichobilharzia* spp. was accompanied by genetic assessment of the snail host, then we could understand more about host-parasite relationship specificity. It could be that there are more species of *Radix* transmitting these species of *Trichobilharzia* than is reported based solely on morphology (e.g. [21, 22, 46]).

Until there is more effort to include multiple and variable gene regions for schistosomes (or any organism) it is not possible to understand the phylogeography or epidemiology of disease-causing helminths. It has been shown repeatedly that variable mitochondrial genes are ideal for assessing cryptic diversity compared to nuclear genes. When only *ITS2* is used without *ITS1*, it is not possible to find enough variation within congeners, particularly if they are closely related. However, a study should not rely only on a single gene (and if it does so, it should be variable and useful for the future), as the diversity revealed is gene diversity, not always directly reflecting species, which must be tested for congruency [50, 55]. The specimens available in GenBank that had variable *cox*1 sequences available represent mostly Western Europe (Table 1). Yet, given the geographic distance between these specimens and Northern Iran, there was very little genetic differentiation in either *cox*1 or *ITS* (Table 5). This result should not be surprising if the long-distance migration of the wild hosts and the suitability of domestic mallards as reservoir hosts are considered in transmission dynamics (also see [4]). Furthermore, Ebbs et al. [25] showed that the intraspecific genetic diversity in *Trichobilharzia querquedulae* was within average range for species schistosomes, even though the comparison included individual worms from across 5 continents.

The occurrence of CD in Iran is high in areas of aquaculture. In addition to wild duck hosts, previous work in the area has shown that domestic mallards are reservoir hosts of *T. regenti*, a nasal schistosome [6], and as well for *T. franki,* shown in this study and thus maintain high prevalence of CD. The genetic results support the finding that populations of *T. franki* from Iran are not differentiated from populations in Europe. Therefore, the schistosomes are dispersed with their migratory duck host, maintaining the gene flow across populations with compatible snail hosts in Iran. It is not surprising that species of *Trichobilharzia* are thought to be one of the common etiological agents of CD; several of these species use snail hosts that are widespread and/or invasive (e.g. [24]) and prefer or at least are easily established in modified aquatic habitats used by domestic animals and humans. Added to this, their definitive hosts travel long distances, further facilitating transmission from one continent to another.

## Conflict of interest

The authors declare that they have no conflict of interest.
